# "Case files from the University of Florida: When an Earache is more than an Earache": A case report

**DOI:** 10.1186/1865-1380-4-33

**Published:** 2011-06-21

**Authors:** Bobby K Desai, Thomas Walls

**Affiliations:** 1 University of Florida, Department of Emergency Medicine, PO BOX 100186, Gainesville, FL, 32610, USA

## Abstract

Brain abscess is not a common diagnosis as there are only approximately 2000 cases reported each year in the United States. There are three main routes of access to the brain including contiguous infection from the oropharynx, direct implantation and hematogenously. We present a case of brain abscess in a child who had multiple visits for ear pain to various physicians including pediatricians and to emergency departments. Additionally, the microbiology of brain abscesses is briefly discussed, as is treatment.

## Introduction

We present a new series for the International Journal of Emergency Medicine, "Case Files from the University of Florida," in which we will present a case seen by the residents and faculty of the Emergency Medicine residency at the University of Florida, Gainesville, and have you, the reader, consider what the diagnostic possibilities are, determine what diagnostic tests are required, and "run" the case. We hope that these cases are educationally rewarding for you.

Presentation

Initial Management

Treatment/Resuscitation

Diagnosis/Disposition

"When an earache is more than an earache"

### Foreword

Patients with otitis media and related conditions present nearly 2 million times to the emergency department every year. The vast majority of these are benign in nature, and the treatment simply observation versus antibiotic therapy. There are occasions, however, where the simple earache turns into something much more. We present such a case.

### Presentation

A 5-year-old child presented to the University of Florida Emergency Department (ED), brought by the mother, with complaints of earache, vomiting, and fever for 3 weeks. The mother had brought the child to their pediatrician the previous week, and he was subsequently diagnosed with dehydration. The parents also brought the child to another emergency department later in the week, and she stated the patient was given intravenous fluids for dehydration. He was discharged home, and his parents given instructions to give acetaminophen for fever and to continue oral rehydration. On this second ED presentation, the mother stated the child was tolerating oral liquids, had urinated once that a.m., and his last bowel movement had been the previous day. The stool was normal in consistency and not bloody. The maximum temperature the patient had was 102°F. The patient had vomited two times on the day of presentation, and it consisted of previously eaten food with no blood. Further history revealed that the child did not attend daycare, there were no smokers in the household, and the child had not received any immunizations for religious reasons. Upon review of systems, the mother denied any rashes, cough, runny nose, complaints of sore throat, diarrhea, or abdominal cramping or pain. She did however state that the patient reported ear pain and facial pain.

Past medical history: None

Past Surgical history: None

Allergies: None

Medications: None

### Physical exam

On presentation the patient's vital signs were: temperature 36.7°C, pulse 60 beats per minute, respiratory rate 28 breaths per minute, and blood pressure 90/39 mmHg. His weight was 22 kg. The patient was alert and looked fatigued, but was conversant with the parents and physician. On eye examination, his pupils were round and reactive to light, without corneal injection. The eyelid exam was normal. The ear exam revealed auricular tenderness of both ears, with bulging tympanic membranes and decreased light reflex. The throat was normal. The lungs were clear to auscultation bilaterally, and the heart exam was unremarkable. He had a soft and non-tender abdomen with normal bowel sounds, and his guaiaic test was negative. His neurological exam was normal.

### Questions to ponder

1. What do you think of this presentation?

2. What differential diagnosis should be considered for this patient?

3. Based on this presentation, what diagnostic tests should be considered?

4. Is anything missing from the history or physical examination?

### Emergency physician's thought process

On initial presentation the patient was afebrile, and his vital signs were stable. He appeared tired and fatigued, but did not appear to be septic. The initial differential diagnosis included an otitis - either media or externa - or perhaps a combination of the two, a simple prolonged upper respiratory infection, Influenza, and viral enteritis. The physicians felt that his emergent condition was due to failed outpatient therapy for vomiting and dehydration. They were concerned about his lack of oral intake, and it was therefore decided to order intravenous fluids. Laboratory tests were also drawn at this time, and these included a chemistry panel and complete blood count. Due to the reported fever, blood cultures were also drawn.

There was no mention in the initial physical examination of mucous membrane moisture or skin turgor, which would be important if the physician was considering a shock-like state for this patient. Additionally, was the patient receiving antipyretics? How much and how often would be important to document. Furthermore, was there any follow-up with the primary care physician after the first ED visit?

One could consider the addition of a urinalysis to evaluate the specific gravity and assess the degree of dehydration, though a BUN/creatinine ratio of 20:1 could detect a pre-renal azotemia.

### Emergency department course

An intravenous line was placed uneventfully, and fluids were started. Laboratory tests were sent and were all within normal limits with the exception of a white blood cell count that was 19,000 cells/mm^3^.

Per physician re-evaluation, the child looked improved, was tolerating oral fluids without difficulty, was afebrile, and his vital signs were normal. He was ambulatory without assistance to the bathroom. However, the family was concerned that these same events occurred at their prior ED visit and requested admission for observation. The ED physician agreed and consulted the pediatric admission team to evaluate the patient. After admission was arranged there was a delay in transporting the patient to the in-patient unit, and he had to remain in the ED until a bed was available.

### Questions to ponder

1. What do you think of this patient's management?

2. Would you add (or remove) any diagnostic tests?

3. Would you change the treatment in any way?

4. Would you have admitted this child?

### Emergency physician's response

Since he appeared "fatigued" based on the physical examination and since his patient's oral intake had diminished over the course of prolonged illness, it seemed reasonable to fluid resuscitate this patient. Since it appeared that he improved over the course of his stay in the ED, this management presumably resulted in the clinical improvement of the patient, since he was now tolerating oral liquids, and due to the patient's unaided ambulation to the bathroom, he acted less fatigued.

The question of whether the patient should have been admitted is a difficult one. The patient seemed to be improved, and looked and presumably felt much better. Based on this clinical gestalt, he did not seem to meet admission criteria. However, it appeared the parents were clearly uncomfortable with his being discharged, and without being privy to the conversation between the emergency physician and family, it is likely a third party - namely the pediatric admitting team - were called to assess the patient. Ultimately, it is a moot point as the admitting team did readily admit the patient, so there clearly was little or no issue in that regard.

### After admission

Four hours after admission, the physician was alerted by the nursing staff that the patient was less alert and lethargic. On examination, he continued to be afebrile - temperature 36.9°C, pulse 72 beats per minute, and respiratory rate 16 breaths per minute. A blood pressure was not recorded. His physical examination revealed an unchanged cardiovascular, pulmonary, and gastrointestinal examination. However, on neurological examination, he was lethargic, and found to have dysarthria and ataxia. A computed tomography (CT) scan was immediately ordered...

### CT scan

**Figure 1:**
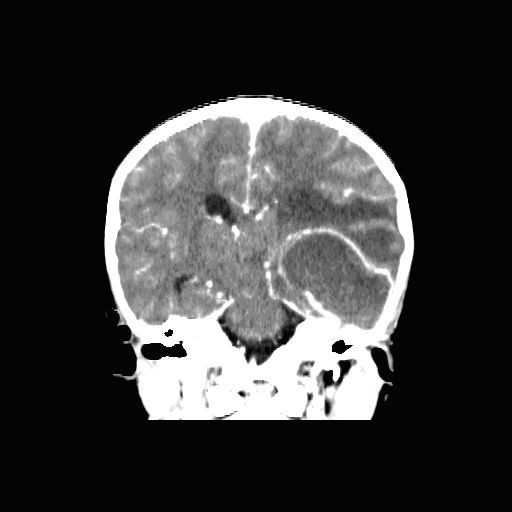
**Coronal view of brain**.

Figure 1 - Coronal view of brain

**Figure 2:**
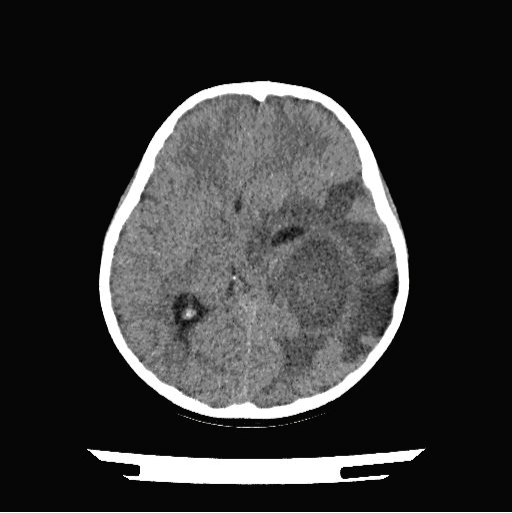
**Transverse view of brain**.

Figure 2 - Transverse view of brain

### Questions to ponder

1. What happened?

2. Could this change have been prevented?

3. What does the CT show?

### Emergency physician's response

This clearly was an acute change in the patient's condition. This most likely could not have been foreseen based on the patient's initial examination, though it can be argued that due to the extent of the edema, a more thorough neurologic examination could have picked up subtle findings. On the other hand, it can be argued that the edema present on the CT scan could have been caused as a result of the fluid resuscitation given to the patient.

The CT showed a 6.1 (anteroposterior) × 4.8 (transverse) × 4.1 (craniocaudal) cm thin ring-enhancing lesion whose epicenter was located in the left low convexity posterior temporal lobe. The lesion is rim-enhancing with a thin peripheral wall, and associated with vasogenic edema. There is 0.9 cm of rightward subfalcine shift, with effacement of the posterior horn of the left lateral ventricle, thus causing sequestration of the ipsilateral temporal horn.

Neurosurgery evaluated the patient and recommended immediate evacuation of the abscess. He was taken to the operating room and had a stereotactic-guided left temporal craniotomy with excision of the brain abscess. He was started on antibiotic therapy and was discharged in good condition 9 days after admission.

## Discussion

Brain abscess is a rare diagnosis; there are only 1,500 to 2,500 reported cases each year in the US [1,2]. Factors that lead to permanent neurologic disability and death due to brain abscess include: impaired host immunity, Glasgow Coma Scale score less than 12, delays in hospitalization, focal neurologic deficits at admission, and uncontrolled diabetes [1-7]. Brain abscess most commonly occurs as the result of contiguous spread of infection from the oropharynx, middle ear, and paranasal sinuses [1,2]. Organisms reach the brain by one of three known routes: hematogenously (one third of cases); from contiguous infections of the middle ear, sinus, or teeth (one third of cases); or by direct implantation by neurosurgery or penetrating trauma (approximately 10% of cases) [8]. The route is unknown in approximately 20% of cases. Circumstances that reduce oxygenation of brain parenchyma are important predisposing factors for bacterial invasion. Spread from a contiguous infection usually involves intervening cerebral thrombophlebitis, with congestive ischemic hypoxemia of the tissue destined to become infected [7,1]. Hematogenous seeding is facilitated by systemic hypoxemia, as in congenital heart diseases with right-to-left shunt and chronic pulmonary suppuration. This is demonstrated by the prominent role of anaerobic bacteria in brain abscesses. The source of brain abscess should be identified for the dual purpose of eliminating the source itself and gaining insight into the probable bacteriologic characteristics of the abscess. Gram-negative rods, especially *Bacteroides*, are the usual pathogens in otogenic brain abscesses, which are typically single and located in the adjacent temporal lobe or cerebellum. Anaerobic and microaerophilic streptococci are the most common pathogens in sinogenic and odontogenic abscesses, and are more typically located in the frontal lobes. Abscesses formed from hematogenous spread are often multiple and polymicrobial, with anaerobic and microaerophilic streptococci commonly represented. Staphylococci are typical pathogens in abscesses due to direct implantation. Gram-negative rods are also suspected in cases related to a neurosurgical procedure. Enteric gram-negative bacilli can be seen in association with an intraabdominal or genitourinary source. *Pseudomonas *spp. can be seen in brain abscesses arising from otitis media or otitis externa [1,2].

In the immunocompromised or elderly patient, opportunistic pathogens must be considered as a potential source of infection. *Nocardia *spp. can be seen from dissemination of cutaneous or pulmonary infection; brain abscesses caused by *M. tuberculosis *and *nontuberculous *mycobacteria have been reported in patients with HIV infection, while *L. monocytogenes *may cause brain abscesses in immunosuppressed individuals [9-11].

Fungal brain abscesses caused by yeast (e.g., *Candida *spp., *Cryptococcus *spp.), dimorphic fungi (e.g., *Histoplasma *spp., *Coccidioides *spp., *Blastomyces *spp.), and molds (e.g., *Aspergillus *spp., *Rhizopus*) are associated with immunocompromised states [1,2]. Zygomycosis can be seen in patients with poorly controlled diabetes [1,2]. Helminths and protozoa can cause parasitic brain abscesses, but these are rare.

### Clinical presentation

Patients with brain abscess may present a myriad of complaints including headache, mental status changes, focal neurologic deficit, fever, and new-onset seizures. Headache and mental status changes are found most frequently, followed by focal neurologic deficits, fever, and seizures [12,13]. The classic clinical triad of fever, headache, and focal neurologic deficits was found to be only 17% sensitive [13,12]. Clinical manifestations are dependent on the location and size of the brain abscess, host immune status, and the virulence of the causative microorganism.

### Diagnosis

CT with intravenous contrast can show ring-enhancing lesions, especially in chronic brain abscesses. However, MRI with gadolinium contrast is more sensitive and specific than CT scan with contrast study to diagnose brain abscess [8]. CT-guided stereotactic biopsy with aspiration of abscesses can reduce the necessity of open craniotomy and can be both diagnostic and therapeutic [14]. It is mandatory to perform microbiologic investigation once the abscess is drained to guide further therapy.

### Treatment

Since brain abscesses are frequently polymicrobial, initial antimicrobial therapy should cover gram-positive, gram-negative, and anaerobic microorganisms, and should be later tailored to the specific organism that is identified [2,3]. The duration of therapy is dependent upon the organism identified; longer therapy is indicated for opportunistic infections, whereas 6-8 weeks of parenteral therapy is indicated for bacterial brain abscesses. Duration of therapy is influenced by causative microorganisms and reduction in the size of the abscess [7,1].

### Follow-Up

Subsequent to the patient's craniotomy and aspiration of contents that morning, his cultures indicated the abscess pathogen to be *Streptococcus pneumoniae*. Blood cultures were negative. He was started on 6 weeks of parenteral therapy. Follow-up 1 month after surgery indicated the child had a mild speech impediment, but was improving. Follow-up 1 year later indicated complete improvement back to his normal neurological function.

## Conclusions

Otitis media and related conditions are a common presenting complaint to the emergency department with over two million visits per year. Treatment failures can potentially occur and the astute clinician must consider other etiologies of otalgia if multiple visits for the same complaint occur. Brain abscess is not a common diagnosis, though potentially has significant morbidity if left undiagnosed. Brain abscess occurs as result of contiguous spread of infection from the oropharynx, middle ear, and paranasal sinuses. Organisms reach the brain hematogenously, contiguous spread from nearby areas or direct implantation. Patients with brain abscess present most commonly with headache and mental status changes. Other common symptoms and signs include focal neurologic deficits, fever and seizures. Contrasted MRI is more sensitive and specific in diagnosing brain abscess than is computed tomography. Treatment is broad spectrum initially, but microbiologic investigation is necessary in order to tailor therapy to the specific cause.

## Consent

Written informed consent was obtained from the parents of the patient for publication of this Case report and any accompanying images. A copy of the written consent is available for review by the Editor-in-Chief of this journal.

## Competing interests

The author declares that they have no competing interests.

## Authors' contributions

TW: Wrote case report. BKD: Formulated questions, answers, and discussion
